# Induction of Labor versus Expectant Management in Women with Preterm Prelabor Rupture of Membranes between 34 and 37 Weeks: A Randomized Controlled Trial

**DOI:** 10.1371/journal.pmed.1001208

**Published:** 2012-04-24

**Authors:** David P. van der Ham, Sylvia M. C. Vijgen, Jan G. Nijhuis, Johannes J. van Beek, Brent C. Opmeer, Antonius L. M. Mulder, Rob Moonen, Mariët Groenewout, Mariëlle G. van Pampus, Gerald D. Mantel, Kitty W. M. Bloemenkamp, Wim J. van Wijngaarden, Marko Sikkema, Monique C. Haak, Paula J. M. Pernet, Martina Porath, Jan F. M. Molkenboer, Simone Kuppens, Anneke Kwee, Michael E. Kars, Mallory Woiski, Martin J. N. Weinans, Hajo I. J. Wildschut, Bettina M. C. Akerboom, Ben W. J. Mol, Christine Willekes, on behalf of the PPROMEXIL trial group

**Affiliations:** 1 Department of Obstetrics and Gynecology, Maastricht University Medical Center, GROW—School for Oncology and Developmental Biology, Maastricht, The Netherlands; 2 Department of Obstetrics and Gynecology, Academic Medical Center Amsterdam, Amsterdam, The Netherlands; 3 Department of Obstetrics and Gynecology, VieCuri Medical Center, Venlo, The Netherlands; 4 Department of Clinical Epidemiology, Biostatistics and Bioinformatics, Academic Medical Center Amsterdam, Amsterdam, The Netherlands; 5 Department of Pediatrics, Maastricht University Medical Center, GROW—School for Oncology and Developmental Biology, Maastricht, The Netherlands; 6 Department of Pediatrics, Atrium Medical Center, Heerlen, The Netherlands; 7 Department of Obstetrics and Gynecology, University Medical Center Groningen, Groningen, The Netherlands; 8 Department of Obstetrics and Gynecology, Isala Klinieken, Zwolle, The Netherlands; 9 Department of Obstetrics and Gynecology, Leiden University Medical Center, Leiden, The Netherlands; 10 Department of Obstetrics and Gynecology, Bronovo Hospital, The Hague, The Netherlands; 11 Department of Obstetrics and Gynecology, Ziekenhuisgroep Twente, Almelo, The Netherlands; 12 Department of Obstetrics and Gynecology, VU University Medical Center, Amsterdam, The Netherlands; 13 Department of Obstetrics and Gynecology, Kennemer Gasthuis, Haarlem, The Netherlands; 14 Department of Obstetrics and Gynecology, Maxima Medical Center, Veldhoven, The Netherlands; 15 Department of Obstetrics and Gynecology, Sint Anna Hospital, Geldrop, The Netherlands; 16 Department of Obstetrics and Gynecology, Catharina Hospital, Eindhoven, The Netherlands; 17 Department of Obstetrics and Gynecology, University Medical Center, Utrecht, The Netherlands; 18 Department of Obstetrics and Gynecology, Sint Antonius Hospital, Nieuwegein, The Netherlands; 19 Department of Obstetrics and Gynecology, Sint Radboud University Medical Center, Nijmegen, The Netherlands; 20 Department of Obstetrics and Gynecology, Gelderse Vallei Hospital, Ede, The Netherlands; 21 Department of Obstetrics and Gynecology, Erasmus Medical Center, Rotterdam, The Netherlands; 22 Department of Obstetrics and Gynecology, Albert Schweitzer Hospital, Dordrecht, The Netherlands; The University of Adelaide, Australia

## Abstract

**Background:**

At present, there is insufficient evidence to guide appropriate management of women with preterm prelabor rupture of membranes (PPROM) near term.

**Methods and Findings:**

We conducted an open-label randomized controlled trial in 60 hospitals in The Netherlands, which included non-laboring women with >24 h of PPROM between 34^+0^ and 37^+0^ wk of gestation. Participants were randomly allocated in a 1∶1 ratio to induction of labor (IoL) or expectant management (EM) using block randomization. The main outcome was neonatal sepsis. Secondary outcomes included mode of delivery, respiratory distress syndrome (RDS), and chorioamnionitis. Patients and caregivers were not blinded to randomization status. We updated a prior meta-analysis on the effect of both interventions on neonatal sepsis, RDS, and cesarean section rate.

From 1 January 2007 to 9 September 2009, 776 patients in 60 hospitals were eligible for the study, of which 536 patients were randomized. Four patients were excluded after randomization. We allocated 266 women (268 neonates) to IoL and 266 women (270 neonates) to EM. Neonatal sepsis occurred in seven (2.6%) newborns of women in the IoL group and in 11 (4.1%) neonates in the EM group (relative risk [RR] 0.64; 95% confidence interval [CI] 0.25 to 1.6). RDS was seen in 21 (7.8%, IoL) versus 17 neonates (6.3%, EM) (RR 1.3; 95% CI 0.67 to 2.3), and a cesarean section was performed in 36 (13%, IoL) versus 37 (14%, EM) women (RR 0.98; 95% CI 0.64 to 1.50). The risk for chorioamnionitis was reduced in the IoL group. No serious adverse events were reported.

Updating an existing meta-analysis with our trial results (the only eligible trial for the update) indicated RRs of 1.06 (95% CI 0.64 to 1.76) for neonatal sepsis (eight trials, 1,230 neonates) and 1.27 (95% CI 0.98 to 1.65) for cesarean section (eight trials, 1,222 women) for IoL compared with EM.

**Conclusions:**

In women whose pregnancy is complicated by late PPROM, neither our trial nor the updated meta-analysis indicates that IoL substantially improves pregnancy outcomes compared with EM.

**Trial registration:**

Current Controlled Trials ISRCTN29313500

*Please see later in the article for the Editors' Summary*

## Abstract 2

In a randomized controlled trial David van der Ham and colleagues investigate induction of labor versus expectant management for women with preterm prelabor rupture of membranes.

## Editors' Summary

**Background:**

Most pregnancies last around 40 weeks, but in industrialized countries, 5%–10% of babies are born before 37 weeks of gestation (gestation is the period during which a baby develops in its mother's womb). Premature birth is a major cause of infant death in many developed countries, and preterm babies can also have short- and/or long-term health problems such as breathing problems, increased susceptibility to life-threatening infections, and learning and developmental disabilities. There are many reasons why some babies are born prematurely, but preterm prelabor rupture of the membranes (PPROM) accounts for 30%–40% of preterm deliveries. Inside the womb, the baby is held in a fluid-filled bag called the amniotic sac. The amniotic fluid cushions the baby, helps some of its organs develop, and protects both mother and baby from infection. The membranes that form the sac usually break at the start of labor (“water breaking”), but in PPROM, the membranes break before the baby is fully grown. PPROM increases the mother's risk of a womb infection called chorioamnionitis and the baby's risk of neonatal sepsis (blood infection), and can trigger early labor.

**Why Was This Study Done?:**

There is currently no consensus on how to manage women whose membranes rupture between 34 and 37 weeks' gestation. Some guidelines recommend immediate induction of labor if PPROM occurs at or beyond 34 weeks' gestation. Others recommend that labor not be induced unless the mother develops signs of infection such as a high temperature or has not delivered her baby spontaneously by 37 weeks' gestation (expectant management). Before 34 weeks' gestation, expectant management is generally recommended. In this randomized controlled trial, the researchers compare the effects of induction of labor and of expectant management on the rate of neonatal sepsis (the proportion of babies that develop neonatal sepsis; the trial's primary outcome) and on secondary outcomes such as the rates of neonatal respiratory distress syndrome (RDS), cesarean section (surgical delivery), and chorioamnionitis in women with PPROM between 34 and 37 weeks' gestation. The researchers also undertake a meta-analysis of published trials on the effect of both interventions on pregnancy outcomes. A randomized controlled trial compares the effects of different interventions in groups of individuals chosen through the play of chance; meta-analysis is a statistical approach that combines the results of several trials.

**What Did the Researchers Do and Find?:**

In the PPROM Expectant Management versus Induction of Labor (PRROMEXIL) trial, 532 non-laboring women with PPROM between 34 and 37 weeks' gestation were randomly assigned to either immediate induction of labor or expectant management. Neonatal sepsis occurred in seven babies born to women in the induction of labor group and in 11 babies born to women in the expectant management group. This difference was not statistically significant. That is, it could have happened by chance. Similarly, although more babies born to women in the induction of labor group than in the expectant management group developed RDS (21 and 17 babies, respectively), this difference was not significant. Cesarean section rates were similar in both intervention groups, but the risk of chorioamnionitis was slightly reduced in the induction of labor group compared to the expectant management group. Finally, the researchers' meta-analysis (which included these new results) found no significant differences in the risk of neonatal sepsis, RDS, or cesarean section associated with the two interventions.

**What Do These Findings Mean?:**

These findings show that, compared to expectant management, induction of labor did not reduce the incidence of neonatal sepsis in pregnancies complicated by PPROM between 34 and 37 weeks' gestation. However, because fewer babies than expected born to the women in the expectant management group developed neonatal sepsis, this trial was underpowered. That is, too few women were enrolled in the trial to enable the detection of a small difference between the interventions in the neonatal sepsis rate. These findings also show that induction of labor did not substantially affect most of the secondary outcomes measured by the researchers. Given these results and those of their meta-analysis, the researchers conclude that, in women whose pregnancy is complicated by PPROM late in pregnancy, induction of labor does not substantially improve the outcome for either the woman or her baby compared to expectant management.

**Additional Information:**

Please access these web sites via the online version of this summary at http://dx.doi.org/10.1371/journal.pmed.1001208.

- The March of Dimes, a nonprofit organization for pregnancy and baby health, provides information on preterm birth (in English and Spanish); its News Moms Need blog contains a post on PPROM
- Tommy's is a nonprofit organization that funds research and provides information on the causes and prevention of miscarriage, premature birth, and stillbirth
- The Royal College of Obstetricians and Gynaecologists guidelines on the diagnosis, investigation, and management of PPROM are available (in English and Russian)
- Information about the PPROMEXIL trial is available
- Personal stories about PPROM are available on the Austprem web site, a non-profit organization that provides information about prematurity and support for parents of premature babies in Australia
- MedlinePlus provides links to other information on premature babies (in English and Spanish)

## Introduction

Preterm prelabor rupture of membranes (PPROM) complicates 1%–5% of all pregnancies and accounts for 30%–40% of all preterm deliveries [1]–[3]. It is associated with increased fetal and maternal morbidity and mortality [4]–[7].

There is no consensus on the management of women with PPROM between 34^+0^ and 37^+0^ wk. The American Congress of Obstetricians and Gynecologists guidelines recommend induction of labor (IoL) if PPROM occurs at or beyond 34^+0^ wk of gestation [8]. The Royal College of Obstetricians and Gynaecologists guidelines state that delivery should be considered at 34^+0^ wk of gestation and recommend that women with PPROM who are managed expectantly beyond 34 wk of gestation be counseled about the increased risk of chorioamnionitis and the presumed decreased risk of neonatal respiratory problems, admission for neonatal intensive care, and cesarean section [9]. The Dutch Society for Obstetrics and Gynecology guidelines advise expectant management (EM) until 35^+0^ wk and recommend discussing IoL with the patient from 35^+0^ wk onwards, whereas IoL is advocated beyond 37^+0^ wk [10]. Canadian and Australian surveys identify a lack of consensus on management in women with PPROM between 34^+0^ and 36^+0^ wk in those countries [11],[12].

A recent Cochrane review on the management of PPROM prior to 37 wk concluded that there is insufficient evidence to guide clinical practice in the management of PPROM [7]. In view of this lack of knowledge, we undertook a randomized controlled trial called PPROM Expectant Management versus Induction of Labor (PPROMEXIL).

In this trial, we tested the hypothesis that IoL reduces neonatal sepsis without increasing neonatal morbidity due to prematurity and without increasing the assisted delivery rate as compared to EM in women with PPROM between 34^+0^ and 37^+0^ wk of gestation.

## Methods

We conducted a multicenter, parallel, open-label randomized controlled trial in The Netherlands, in which all eight academic and 52 non-academic hospitals participated. The study was approved by the medical ethics committee of the Maastricht University Medical Center, Maastricht, The Netherlands (Ref. no. MEC 05-240, 8 March 2006). Local approval was given by the boards of each of the participating hospitals. After the start of the trial, no changes were made to the trial protocol or to the trial outcome measures. The protocol has been published previously [13]. The trial was registered in the ISRCTN register (ISRCTN29313500; Text S1). This trial is reported in concordance with the CONSORT 2010 checklist (Text S2).

### Patients

Women with a singleton or twin pregnancy presenting with PPROM between 34^+0^ and 36^+6^ wk of gestation who were not in labor within 24 h after rupture of membranes were eligible for this study. Women in whom PPROM was diagnosed after 26^+0^ wk, but who had not delivered by 34^+0^ wk of gestational age were also eligible for participation. All eligible patients were counseled about participation in the study within the first 24 h after PPROM had occurred. Informed consent was obtained from all who participated in the study at least 24 h after PPROM, as soon as was possible and convenient. Breech presentation was not an exclusion criterion, as both cesarean and vaginal delivery were allowed in the protocol. Women with a monochorionic multiple pregnancy; abnormal (non-reassuring) cardiotocogram; meconium-stained amniotic fluid; signs of intrauterine infection; major fetal anomalies; hemolysis, elevated liver enzymes, and low platelets (HELLP syndrome); or severe preeclampsia were not eligible for the study. Women who declined consent for randomization but authorized use of their medical data were included in the database and were followed in the patient preference arm.

Rupture of membranes was diagnosed based on history and clinical findings such as gross vaginal fluid loss, in combination with other available diagnostic test methods when necessary. The final decision on whether or not a patient had rupture of membranes was made by the attending staff.

Gestational age was based either on first trimester ultrasound scan or, in women with a regular cycle, on the first day of the last menstrual period if the expected date of delivery differed less than 7 d from that estimated by ultrasound. In women with an unknown/uncontrolled pregnancy beyond the first trimester, gestational age was estimated by second trimester ultrasound measurements.

### Randomization

After written informed consent had been obtained, patient data were entered in a password-protected web-based database. Randomization was performed on a central password-protected web-based application developed by the clinical trial unit of the Academic Medical Center Amsterdam, Amsterdam, The Netherlands. The randomization sequence was created using a block size of four, stratified for center and parity, in a 1∶1 ratio for immediate IoL versus EM.

### Procedures

#### Induction of labor

Patients allocated to IoL were induced within 24 h after randomization. Induction was performed according to the national guidelines [14]. After vaginal examination labor was induced with either prostaglandin or oxytocin. In the case of planned cesarean section, the cesarean section was performed as soon as feasible after randomization.

#### Expectant management

Women randomized to EM were monitored according to local protocol until spontaneous delivery, which could be in an outpatient or an inpatient setting. Monitoring in both settings consisted of at least daily maternal temperature monitoring and twice weekly blood sampling for maternal leukocyte count and C-reactive protein measurement. When a patient in the EM group reached 37^+0^ wk of gestational age, labor was induced according to the national guidelines [10]. Whenever a patient with an indication for planned cesarean section was allocated to EM, the cesarean section was performed as soon as labor commenced. Labor was induced prior to 37^+0^ wk of gestation if there were clinical signs of infection or when another fetal or maternal indication occurred that warranted IoL.

### Data Collection

At all local centers, data collection was the responsibility of the local investigators, who were supported by regional research nurses and midwives. Data were collected, coded, and processed with adequate precautions to ensure patient confidentiality.

At study entry, baseline demographics and obstetric and medical history were recorded. Ethnicity and country of birth were recorded by patients themselves. Baseline blood samples were taken from all participating women at study entry. A vaginal swab was collected either at admission to the hospital or at study entry. The national guidelines of the Dutch Society for Obstetrics and Gynecology do not indicate whether to start or not start antibiotics in women with PPROM prior to 37 wk [10]. Thus, antepartum administration of antibiotics was given according to local protocol.

Postpartum neonatal and maternal outcome measures were recorded, including maternal and neonatal length of stay in hospital. The placenta was sent for histological examination to determine the presence or absence of chorioamnionitis, and funisitis in particular. Serious adverse events were reported to the Adverse Events Committee of the study. No interim analysis was planned, as both treatment options were common practice.

### Outcome Measures

#### Primary outcome

Neonatal sepsis was defined as follows: (1) blood culture taken at birth found positive for bacteria (excluding *Staphylococcus epidermidis*) or (2) two or more symptoms of infection (apnea, temperature instability, lethargy, feeding intolerance, respiratory distress, hemodynamic instability) within 72 h after birth, plus one of the following: (a) positive blood culture (culture-proven sepsis); (b) C-reactive protein >20 mmol/l (suspicion of sepsis); (c) positive surface cultures of a known virulent pathogen (suspicion of sepsis).

When a local investigator classified a case as sepsis, or when criteria for sepsis were entered in the database, the case was judged by an independent panel of pediatricians (A. L. M. M. and R. M.) who, unaware of the allocation of randomization, adjudicated between neonatal sepsis (proven or suspected sepsis) or no sepsis [13].

#### Secondary outcomes

Secondary neonatal outcome measures were respiratory distress syndrome (RDS), wet lung, meconium aspiration syndrome, pneumothorax/pneumomediastinum, asphyxia, late onset neonatal sepsis, hypoglycemia, necrotizing enterocolitis, hyperbilirubinemia, intraventricular hemorrhage, periventricular leucomalacia, convulsions, other neurological abnormalities, other complications, intrapartum death, total length of hospital stay and admission, and length of stay on neonatal intensive care unit (NICU).

Maternal outcome measures were antepartum hemorrhage, uterine rupture, umbilical cord prolapse, signs of chorioamnionitis (defined as fever before or during labor as a temperature greater than 37.5°C on two occasion more than 1 h apart before or during labor, or a temperature greater than 38.0°C on one occasion with uterine tenderness, leukocytosis, maternal or fetal tachycardia, or a foul-smelling vaginal discharge in absence of any other cause of hyperpyrexia), maternal sepsis (defined as a temperature greater than 38.5°C and a positive blood culture or circulatory instability requiring intensive care monitoring), thromboembolic complications, urinary tract infection treated with antibiotics, endometritis (defined as a temperature greater than 38.0°C on two occasions at least 1 h apart after the first 24 h postpartum with associated uterine tenderness), pneumonia, anaphylactic shock, HELLP syndrome, maternal death, other complications, total length of hospital stay, and admission to the intensive care unit. Finally, we recorded mode of delivery and need for anesthesia [13].

### Statistical Analysis

#### Sample size

The proportion of neonates with sepsis was hypothesized to be 7.5% in the EM group and 2.5% in the IoL group. Because it was considered clinically not plausible that IoL would lead to a higher proportion of neonatal sepsis as compared to EM, we performed a power analysis based on a one-sided test, without continuity correction. This required 260 women per treatment arm to statistically demonstrate a 66% risk reduction with 80% power and a 5% type one error probability.

#### Data analysis

Data were analyzed on an intention to treat basis. After tabulation, study baseline characteristics were compared. Continuous data were tested with the Student's *t* test or the non-parametric Mann-Whitney U test. Relative risks (RRs), mean differences, and 95% confidence intervals (CIs) were calculated for the relevant outcome measures. Categorical data were analyzed with χ^2^ statistics. Since the randomization was stratified for center and parity, we performed a stratified analysis using a Cochran-Mantel-Haenszel correction. The primary outcome of neonatal sepsis is presented as RR after applying the Cochran-Mantel-Haenszel correction. Kaplan-Meier curves were constructed to analyze time from randomization to delivery in both study arms. These curves were compared using the log rank test. *p*-Values below 0.05 were considered to indicate statistical significance. Statistical analyses were performed using SPSS Statistics (version 17.0).

### Meta-Analysis

We updated a recent Cochrane review [7] on sepsis, RDS, and cesarean section rate. To do so, we performed an additional search in MEDLINE and CENTRAL (from 1 October 2009 until 30 April 2011), using the same strategy as described by Buchanan et al. in order to find additional papers that were not in the systematic review [7]. Two authors (D. P. v. d. H. and B. W. J. M.) identified papers for relevance and quality, and extracted the data. We calculated risk ratios, with 95% CIs for all outcomes. Buchanan et al. subdivided their analysis between overall sepsis (defined or undefined by the authors) and culture-proven sepsis [7]. They also made a comparison between suspicion of neonatal sepsis and management of labor. In this comparison they included one study [15]. Because of the broad definition given by the authors of this study for suspicion of sepsis (“clinical findings suggestive for neonatal sepsis”), we considered our definition of suspicion of neonatal sepsis not comparable. But we considered our overall sepsis rate as comparable with the overall sepsis rate, and added the culture-proven sepsis cases in our study to the culture-proven sepsis comparison in the meta-analysis.

Statistical analyses were carried out using RevMan, version 5.1 [16].

## Results

**Figure 1:**
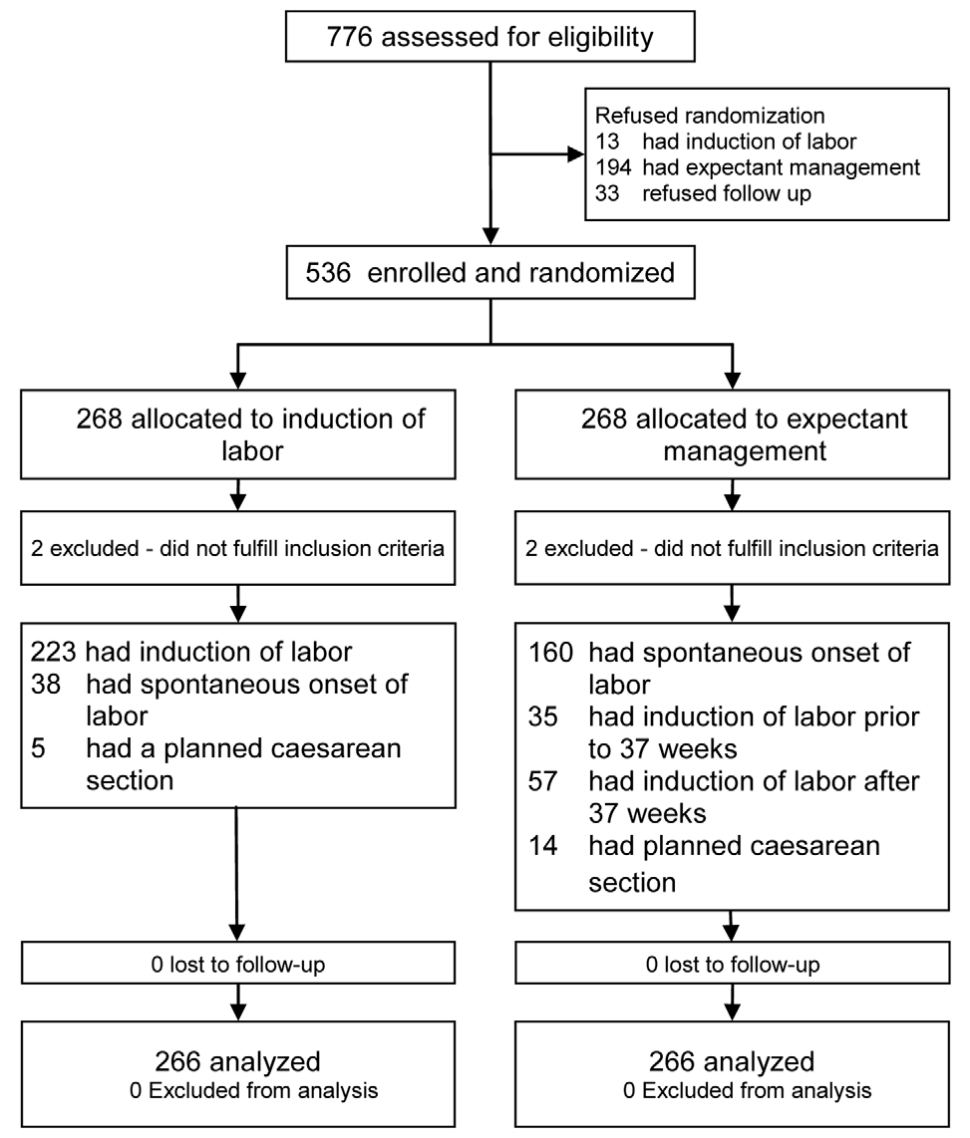
Trial profile.

From 1 January 2007 until 9 September 2009 a total of 776 women were asked to participate in the trial, of which 536 women (69%) gave informed consent. A total of 268 women were randomized to IoL (IoL group) and 268 to EM (EM group). Figure 1 outlines the study profile. In both arms two patients were excluded because after completion of the trial it became clear that their gestational age was over 36^+6^ wk at the time of inclusion. Baseline characteristics were comparable between the two groups (Table 1). Median gestational age at randomization was 251 d (35^+6^ wk). The percentage of women that had PPROM before 34 wk of gestation was 14% in both groups.

**Table 1:** Baseline characteristics.

Characteristic^a^	IoL (*n* = 266)	EM (*n* = 266)
**Maternal age (range) [±SD], y**	29.5 (18.3–43.3) [±4.9]	29.6 (18.1–46.7) [±5.6]
**Number nulliparous (parity range) (percent nulliparous)**	147 (0–5) (55%)	152 (0–5) (57%)
**Twin pregnancy**	2 (0.8%)	4 (1.5%)
**Ethnic origin**		
White	211 (79%)	209 (79%)
Other ethnic origin	35 (13%)	45 (17%)
Unknown	20 (7.5%)	14 (5.3%)
**Education** b **^,^** c		
Primary school (4 to 12 y)	10 (6.6%)	0 (0%)
Secondary school (12–18 y)	11 (7.2%)	15 (9.5%)
Lower professional school	14 (9.2%)	15 (9.5%)
Medium professional school	69 (46%)	80 (51%)
Higher professional school	35 (23%)	34 (22%)
University	12 (7.9%)	14 (8.9%)
**Maternal smoking**	58 (23%)	63 (25%)
**Antenatal administration of corticosteroids**	37 (15%)	39 (15%)
**Body mass index** c		
At booking (range) [±SD], kg/m^2^	24.8 (17.0–52.2) [±5.7]	24.6 (16.4–45.1) [±5.1]
At study entry (range) [±SD], kg/m^2^	29.4 (16.3–52.1) [±6.3]	28.7 (17.9–46.3) [±5.7]
**Diagnostic test for rupture of membranes** d		
Positive history	224 (84%)	235 (88%)
Positive ferning	127 (48%)	133 (50%)
Positive pH test	9 (3.4%)	10 (3.8%)
Positive PAMG-1 test	17 (6.4%)	18 (6.8%)
Other positive ROM test	18 (6·8%)	10 (3·8%)
Decrease amniotic fluid on ultrasound	126 (47%)	133 (50%)
**Gestational age at PPROM**		
<34 wk	36 (14%)	38 (14%)
34^+0^ to 34^+6^ wk	41 (15%)	35 (13%)
35^+0^ to 35^+6^ wk	79 (30%)	84 (32%)
36^+0^ to 36^+6^ wk	110 (41%)	109 (41%)
**Gestational age at PPROM, median [IQR], d**	249 [243–253]	249 [243–253]
**Gestational age at randomization, median [IQR], d**	251 [245–255]	251 [245–255]
**Fetal position at data entry**		
Cephalic	251 (94%)	245 (92%)
Breech	15 (5.6%)	21 (7.9%)
**Maternal temperature at inclusion, mean [±SD], °C** c	36.9 [±0.48]	36.9 [±0.44]

Data are presented as number (percent) unless otherwise indicated.

a Percents given are related to available data per characteristic and may differ from total number of patients.

b Percents given as part of known educational level.

c Outcome characteristic with more than 5% missing data. Education: data available for 310 women (58%); body mass index at booking: data available for 453 women (85%); body mass index at start study available for 266 women (50%); maternal temperature at inclusion: data available for 504 women (95%).

d Sum of tests exceeds 100% because more than one test could be applied on the same patient.

IQR, interquartile range; PAMG-1, placental alpha macroglobulin-1; ROM, rupture of membranes; SD, standard deviation.

**Figure 2:**
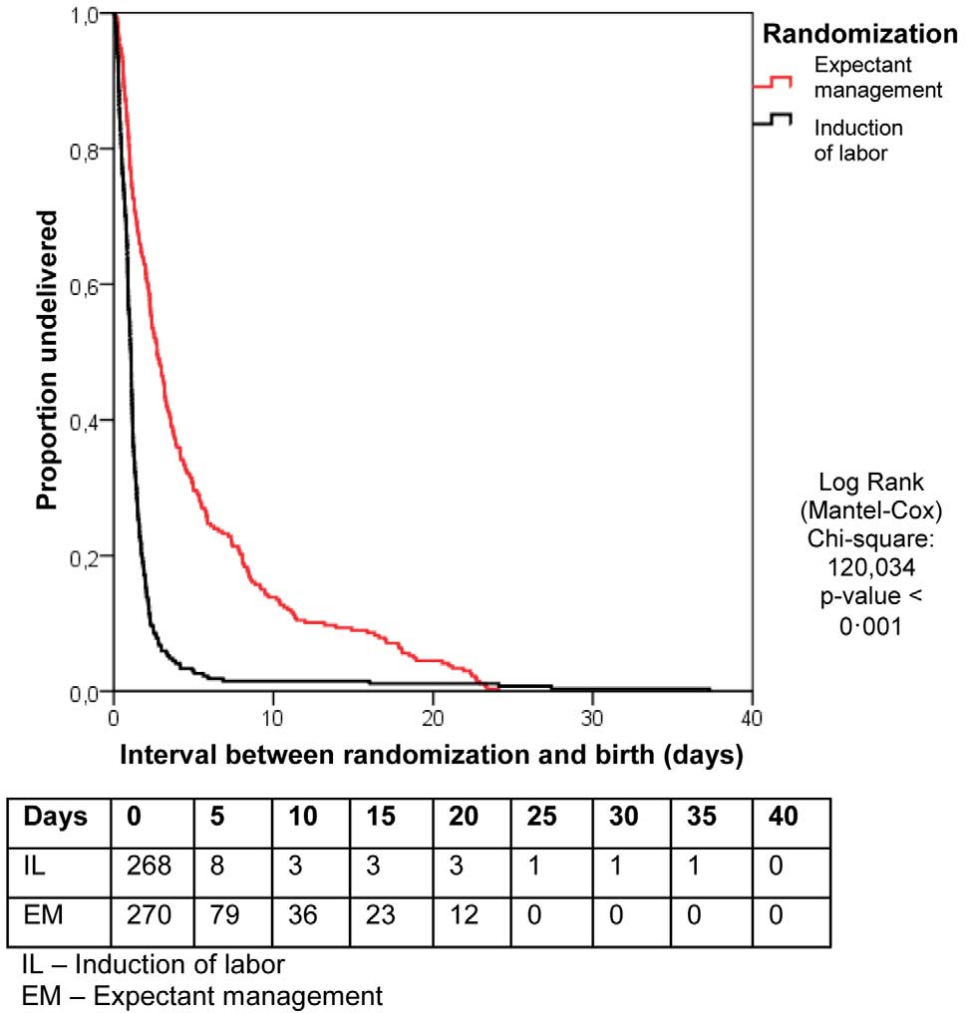
Kaplan-Meier curve for the interval between randomization and birth.

Table 2 shows the data on pregnancy outcome and mode of delivery. In the EM group, labor was induced in 94 women (34%), in 70 (77%) of these cases because the gestational age of 37 wk was reached. In 24 women induction was prior to 37 wk, in four cases (4.4%), this was for fetal distress, in four (4.4%) for meconium-stained amniotic fluid, in six (6.6%) for signs of infections, in three (3.3%) for maternal hypertensive disorders, and in two (2.2%) for other maternal complications. In the remaining two patients (2.2%), no reason for induction was recorded. Figure 2 shows the Kaplan-Meier curve for the interval between randomization and delivery in both groups.

**Table 2:** Pregnancy outcomes.

Outcome^a^	IoL (*n* = 266/268)^b^	EM (*n* = 266/270)^c^	RR or Mean Difference (95% CI; *p*-Value)	Absolute Risk Reduction (95% CI)
**Onset of labor**				
Spontaneous	38 (14%)	161 (61%)	0.24 (0.17 to 0.32; <0.0001)	46.3% (39.0% to 53.5%)
Planned cesarean section	5 (1.9%)	14 (5.3%)	0.36 (0.13 to 0.98; 0.036)	3.3% (0.24% to 6.5%)
Induction	223 (84%)	91 (34%)	2.45 (2.06 to 2.92; <0.0001)	−49.6% (−42.4% to −56.8%)
≥37 wk gestational age	0 (0%)	70 (77%)	NA	NA
**Gestational age at birth, mean [±SD] (median) [IQR], d**	250.9 [±6.6] (252) [246–256]	254.3 [±5.8] (256) [251–259]	−3.34 (−4.39 to −2.29; <0.0001)^d^	NA
**Gestational age at birth**				
34^+0^ to 34^+6^ wk	49 (18%)	22 (8.1%)	2.24 (1.0 to 3.60; 0.0005)	−10.1% (−4.47% to −15.8%)
35^+0^ to 35^+6^ wk	79 (30%)	49 (18%)	1.62 (1.19 to 2.22; 0.002)	−11.3% (−4.19% to −18.5%)
36^+0^ to 36^+6^ wk	124 (46%)	110 (41%)	1.14 (0.94 to 1.38; 0.196)	−5.53% (−13.9% to 2.84%)
37^+0^ to 37^+6^ wk	14 (5.2%)	89 (33%)	0.16 (0.09 to 0.27; <0.0001)	27.7% (21.5% to 34.0%)
>38 wk	2 (0.7%)^e^	0 (0%)	0.155	−0.75% (−1.77% to 0.28%)
**Interval between randomization and birth, mean [±SD] (median) [IQR], h**	38.4 [±79.7] (26) [14–38]	117 [±135] (65) [26–141]	−78.5 (−97.3 to −59.7; <0.0001)^d^	NA
**Interval between rupture of membranes and birth, mean [±SD] (median) [IQR], h**	103 [±140] (62) [47–98]	202 [±234] (110) [64–234]	−98.3 (−131 to −65.2; <0.0001)^d^	NA
**Mode of delivery**				
Vaginal, spontaneous	213 (80%)	209 (77%)	1.03 (0.94 to 1.12; 0.559)	−2.07% (−9.02% to 4.88%)
Vaginal, assisted	19 (7.1%)^f^	24 (8.9%)^g^	0.80 (0.45 to 1.42; 0.442)	1.80% (−2.78% to 6.38%)
Cesarean section	36 (13%)	37 (14%)	0.98 (0.64 to 1.50; 0.927)	0.27% (−5.52% to 6.06%)
Any instrumental delivery	55 (21%)	61 (23%)	0.91 (0.66 to 1.26; 0.559)	2.07% (−4.88% to 9.02%)
**Antibiotics**				
During admission	92 (35%)	92 (35%)	1.00 (0.79 to 1.26; >0.999)	0.0% (−8.08% to 8.08%)
During labor	84 (32%)	73 (28%)	1.14 (0.88 to 1.48; 0.336)	−3.85% (−11.7% to 3.98%)
During admission or labor	112 (42%)	108 (41%)	1.04 (0.85 to 1.27; 0.725)	−1.50% (−9.87% to 6.86%)
Epidural and/or spinal analgesia	71 (27%)	43 (16%)	1.65 (1.18 to 2.32; 0.003)	−10.5% (−17.4% to −3.61%)
**Hemorrhage, mean (range) [±SD], ml**	420 (50–5,000) [±471]	437 (50–4,000) [±491]	−17.8 (−101 to 65.1; 0.674)	NA
**Total maternal admission, mean [±SD] (median) [IQR], d**	9.3 [±6.2] (8) [6–12]	11.3 [±8.3] (9) [6–14]	−1.94 (−3.21 to −0.68; 0.003)	NA

Data are presented as number (percent) unless otherwise indicated.

a Percents, RRs, 95% CIs, and *p*-values given are related to available data per characteristic and may differ from the total number of patients.

b The number of women in the IoL group was 266; the number of newborns in the IoL group was 268.

c The number of women in the EM group was 266; the number of newborns in the EM group was 270.

d Mean difference with 95% CI.

e After randomization, the diagnosis rupture of membranes was reconsidered for these two women, and they were managed as having intact membranes.

f Including one forceps extraction.

g Including two forceps extractions.

IQR, interquartile range; NA, not applicable; SD, standard deviation.

Of the 266 women allocated to the IoL group, 38 (14%) went into labor spontaneously (i.e., after randomization but before it was possible to start induction), five (2%) had a planned cesarean section, and in the remaining 223 (84%) women, labor was induced.

Women allocated to IoL obtained epidural or spinal analgesia more often than those in the EM group, 71 (27%) versus 43 (16%), respectively (RR 1.7; 95% CI 1.2 to 2.3). There was no difference in antibiotic administration during admission or labor between the two groups. The cesarean section rate was comparable in both groups (36 [13%] cesarean sections in the IoL group versus 37 [14%] in the EM group; RR 0.98; 95% CI 0.64 to 1.5). No serious adverse events were reported during the study period.

### Neonatal Sepsis

Neonatal sepsis was seen in seven newborns (2.6%) in the IoL group versus 11 (4.1%) in the EM group (RR 0.64; 95% CI 0.25 to 1.6) (see Table 3). The RR for neonatal sepsis was similar when stratified for center and parity (RR 0.65; 95% CI 0.25 to 1.7).

**Table 3:** Neonatal outcomes.

Outcome^a^	IoL (*n* = 268)	EM (*n* = 270)	RR or Mean Difference (95% CI; *p*-Value)	Absolute Risk Reduction (95% CI)
**Primary outcome**				
Proven neonatal sepsis	1 (0.4%)	3 (1.1%)	0.34 (0.04 to 3.21; 0.319)	0.74% (−0.71% to 2.19%)
Suspected neonatal sepsis	6 (2.2%)	8 (2.9%)	0.76 (0.27 to 2.15; 0.598)	0.72% (−1.96% to 3.41%)
Sepsis overall	7 (2.6%)	11 (4.1%)	0.64 (0.25 to 1.63; 0.346)	1.46% (−1.57% to 4.50%)
**Secondary outcomes**				
Apgar score at 1 min <7	12 (4.5%)	17 (6.4%)	0.70 (0.34 to 1.44; 0.340)	1.87% (−1.97% to 5.71%)
Apgar score at 5 min <7	2 (0.7%)	1 (0.4%)	2.02 (0.18 to 22.1; 0.558)	−0.38% (−1.64% to 0.89%)
Neonatal temperature >38.0°C^b^	16 (11%)	6 (4.1%)	2.74 (1.10 to 6.81; 0.022)	−7.06% (−13.1% to −1.02%)
pH umbilical artery <7.1 mmol/l^b^	9 (4.6%)	5 (2.5%)	1.87 (0.64 to 5.47; 0.249)	−2.14% (−5.78% to 1.50%)
Birth weight, mean [±SD], g	2,660 (±438)	2,723 (±414)	−62.7 (−135 to 9.44; 0.088)^c^	NA
Respiratory distress	21 (7.8%)	17 (6.3%)	1.25 (0.67 to 2.31; 0.486)	−1.54% (−5.57% to 2.79%)
Wet lung	2 (0.7%)	4 (1.5%)	0.50 (0.09 to 2.73; 0.417)	0.74% (−1.04% 2.51%)
Asphyxia	0 (0%)	0 (0%)	NA	NA
Pneumothorax/pneumomediastinum	0 (0%)	1 (0.4%)	0.322	0.38% (−0.37% to 1.13%)
Meconium aspiration syndrome	0 (0%)	0 (0%)	NA	NA
Neonatal meningitis	0 (0%)	1 (0.4%)	0.323	0.38% (−0.37% to 1.14%)
Late onset sepsis	0 (0%)	1 (0.4%)	0.323	0.38% (−0.37% to 1.13%)
Hypoglycemia	49 (19%)	23 (8.9%)	2.16 (1.36 to 3.43; 0.0008)	−10.3% (−16.2% to −4.33%)
Hyperbilirubinemia	96 (38%)	67 (26%)	1.47 (1.13 to 1.90; 0.004)	−11.9% (−19.9% to −3.97%)
Necrotizing enterocolitis	0 (0%)	0 (0%)	NA	NA
HIE grade 1 or 2	0 (0%)	0 (0%)	NA	NA
HIE grade 3 or 4	0 (0%)	0 (0%)	NA	NA
IVH grade 1 or 2^b^	0 (0%)	1 (0.4%)	0.325	0.39% (−0.37% to 1.15%)
IVH grade 3 or 4^b^	0 (0%)	1 (0.4%)	0.325	0.39% (−0.37% to 1.15%)
PVL grade 1 or 2	1 (0.4%)	0 (0%)	0.314	−0.40% (−1.17 to 0.38%)
PVL grade 3 or 4	0 (0%)	0 (0%)	NA	NA
Convulsions	0 (0%)	1 (0.4%)	0.322	0.38% (−0.36% to 1.13%)
Other neurologic disorders	2 (0.8%)	3 (1.2%)	0.68 (0.11 to 4.02; 0.666)	0.37% (−1.32% to 2.06%)
Other disorders	25 (9.8%)	37 (15%)	0.68 (0.42 to 1.09; 0.104)	4.69% (−0.95% to 10.3%)
Intrapartum death	0 (0%)	0 (0%)	NA	NA
Neonatal death	0 (0%)	0 (0%)	NA	NA
Hospital admission	251 (94%)	253 (94%)	0.999 (0.96 to 1.05; 0.98)	0.05% (4.1% to 4.2%)
Length of hospital stay, mean [±SD] (median) [IQR], d	8.0 [±7.1] (6) [2.5–11]	6.5 [±7.9] (4) [2–9]	1.4 (0.11 to 2.74; 0.034)^c^	NA
NICU admission	24 (9.0%)	15 (5.6%)	1.61 (0.86 to 3.00; 0.128)	−3.40% (−7.78% to 0.98%)
Length of NICU stay, mean [±SD] (median) [IQR], d	4.1 [±4.1] (2) [1–6]	8.1 [±7.9] (5) [3–12]	−3.98 (−7.89 to −0.08; 0.046)^c^	NA

Data are presented as number (percent) unless otherwise indicated.

a Percentages, RRs, 95% CIs, and *p*-values given according to available data.

b Outcome characteristic with more than 5% missing data. Neonatal temperature data available for 292 infants (54%); pH umbilical artery data available for 397 (74%); intraventricular hemorrhage data available for 508 (94%).

c Mean difference with 95% CI.

HIE, hypoxic ischemic encephalopathy; IQR, interquartile range; IVH, intraventricular hemorrhage; NA, not applicable; PVL, periventricular leucomalacia; SD, standard deviation.

### Other Neonatal Outcomes

Table 3 shows all neonatal outcomes. Neonates born in the IoL group stayed 1.4 d longer in hospital than neonates born after EM and were admitted more often to the NICU. Newborns admitted to the NICU in the IoL group stayed shorter than those in the EM group.

RDS was seen in 21 (7.8%) newborns in the IoL group versus 17 (6.3%) in the EM group. Hypoglycemia (RR 2.2) and hyperbilirubinemia (RR 1.5) were seen significantly more often in the IoL group. For other neonatal outcome measures, there were no significant differences between the two groups. Seventy-six newborns (28%) in the IoL group were treated with antibiotics, for an average of 5.0 d, versus 78 (27%) in the EM group, for an average of 5.0 d (Table 4).

**Table 4:** Neonatal treatments.

Outcome^a^	IoL (*n* = 268)	EM (*n* = 270)	RR or Mean Difference (95% CI; *p*-Value)	Absolute Risk Reduction (95% CI)
**Antibiotic treatment, number (percent)**				
Augmentin	12 (4.5%)	18 (6.7%)	0.67 (0.33 to 1.37; 0.270)	2.19% (−1.68% to 6.06%)
Amoxicillin	49 (18%)	55 (20%)	0.90 (0.64 to 1.27; 0.540)	2.09% (−4.58% to 8.76%)
Gentamycin	44 (16%)	45 (17%)	0.99 (0.67 to 1.44; 0.938)	0.25% (−6.03% to 6.53%)
Cephalosporin	10 (3.7%)	9 (3.3%)	1.12 (0.46 to 2.71; 0.803)	−0.40% (−3.52% to 2.72%)
Other antibiotics	25 (9.3%)	19 (7.0%)	1.33 (0.75 to 2.35; 0.332)	−2.29% (−6.92% to 2.34%)
Any antibiotic treatment	76 (28%)	75 (28%)	1.02 (0.78 to 1.34; 0.881)	−0.58% (−8.17% to 7.01%)
**Antibiotic treatment length, mean (±SD)** b				
Augmentin	4.5 (±2.3)	5.2 (±2.5)	−0.72 (−2.57 to 1.13; 0.430)^c^	NA
Amoxicillin	4.8 (±2.8)	5.2 (±2.5)	−0.46 (−1.50 to 0.57; 0.377)^c^	NA
Gentamycin	4.4 (±1.9)	3.7 (±1.6)	0.68 (−0.07 to 1.42; 0.076)^c^	NA
Cephalosporin	4.7 (±3.8)	5.4 (±2.2)	−0.74 (−3.83 to 2.34; 0.617)^c^	NA
Other antibiotics	4.8 (±2.7)	5.1 (±2.7)	−0.25 (−1.97 to 1.46; 0.768)^c^	NA
Any antibiotic treatment	5.1 (±2.8)	5.1 (±2.5)	−0.01 (−0.86 to 0.84; 0.974)^c^	NA
**Other neonatal treatment, number (percent)**				
Positive pressure ventilation with endotracheal tube	2 (0.7%)	4 (1.5%)	0.50 (0.09 to 2.73; 0.417)	0.73% (−1.04% to 2.51%)
Positive pressure ventilation	11 (4.1%)	9 (3.3%)	1.23 (0.52 to 2.92; 0.636)	−0.77% (−3.97% to 2.43%)
Tube feeding	37 (14%)	33 (12%)	1.13 (0.73 to 1.75; 0.585)	−1.58% (−7.27% to 4.10%)
Total parenteral feeding	5 (1.9%)	5 (1.9%)	1.007 (0.30 to 3.44; 0.991)	−0.01% (−2.29% to 2.27%)
**Other neonatal treatment length, mean (±SD)** d				
Positive pressure ventilation with endotracheal tube	2.0 (±1.4)	2.5 (±1.3)	−0.50 (−3.68 to 2.68; 0.685)^c^	NA
Positive pressure ventilation	3.1 (±2.9)	1.8 (±0.97)	1.40 (−0.77 to 3.58; 0.191)^c^	NA
Tube feeding	8.1 (±4.5)	6.0 (±4.2)	2.16 (0.07 to 4.25; 0.043)^c^	NA
Total parenteral feeding	5.2 (±2.7)	7.2 (±9.8)	2.00 (−12.5 to 8.51; 0.672)^c^	NA

a Percentages given according to available data.

b Mean treatment length calculated for neonates receiving each antibiotic.

c Mean difference with 95% CI.

d Mean treatment length calculated from neonates receiving each treatment.

NA, not applicable; SD, standard deviation.

### Maternal Outcomes

Table 5 shows the maternal outcomes. Clinical chorioamnionitis was seen in six women (2.3%) in the IoL group versus 15 (5.6%) women in the EM group (RR 0.40; 95% CI 0.16 to 1.02). The incidence of histological chorioamnionitis was 43 (22%) versus 62 (32%), respectively (RR 0.69; 95% CI 0.49 to 0.96).

**Table 5:** Maternal outcomes.

Outcome^a^	IoL (*n* = 266)	EM (*n* = 266)	RR (95% CI; *p*-Value)	Absolute Risk Reduction (95% CI)
**Maternal complications**				
Antepartum hemorrhage	2 (0.8%)	5 (1.9%)	0.40 (0.08 to 2.04; 0.255)	1.13% (−0.81% to 3.06%)
Cord prolapse	1 (0.4%)	0 (0%)	0.319	−0.38% (−1.11% to 0.36%)
Uterine rupture	1 (0.4%)	0 (0%)	0.319	−0.38% (−1.11% to 0.36%)
Clinical chorioamnionitis	6 (2.3%)	15 (5.6%)	0.40 (0.16 to 1.02; 0.045)	3.38% (0.09% to 6.68%)
Sepsis	6 (2.3%)	1 (0.4%)	6.00 (0.72 to 49.5; 0.057)	−1.88% (−3.81% to 0.05%)
Thromboembolic complications	0 (0%)	1 (0.4%)	0.319	0.38% (−0.36% to 1.11%)
Urinary tract infections treated with antibiotics	4 (1.5%)	0 (0%)	0.045	−1.50% (−2.97% to −0.04%)
Endometritis	2 (0.8%)	4 (1.5%)	0.50 (0.09 to 2.71; 0.412)	0.75% (−1.04% to 2.55%)
Pneumonia	0 (0%)	1 (0.4%)	0.319	0.38% (−0.36% to 1.11%)
Anaphylactic shock	0 (0%)	0 (0%)	NA	NA
HELLP syndrome	0 (0%)	2 (0.8%)	0.157	0.75% (−0.29% to 1.79%)
Death	0 (0%)	0 (0%)	NA	NA
Other complications	11 (4.1%)	9 (3.4%)	1.22 (0.52 to 2.90; 0.649)	−0.75% (−3.98% to 2.48%)
**Perineum**				
No laceration	116 (44%)	114 (43%)	1.02 (0.84 to 1.24; 0.831)	−0.92% (−9.35% to 7.51%)
First degree laceration	49 (19%)	52 (20%)	0.95 (0.67 to 1.34; 0.756)	1.06% (−5.62% to 7.73%)
Second degree laceration	27 (10%)	35 (13%)	0.77 (0.48 to 1.24; 0.288)	2.97% (−2.49% to 8.43%)
Third degree laceration	2 (0.8%)	2 (0.8%)	1.004 (0.14 to 7.07; 0.997)	0.003% (−1.47% to 1.47%)
Fourth degree laceration	2 (0.8%)	4 (1.5%)	0.50 (0.09 to 2.72; 0.414)	0.75% (−1.05% to 2.55%)
Episiotomy	69 (26%)	59 (22%)	1.17 (0.87 to 1.59; 0.301)	−3.86% (−11.1% to 3.41%)
**Delivery of placenta**				
Spontaneous	211 (79%)	209 (79%)	1.01 (0.92 to 1.10; 0.832)	−0.75% (−7.68% to 6.18%)
Manual placental removal	19 (7.1%)	20 (7.5%)	0.95 (0.52 to 1.74; 0.868)	−0.38% (−4.05% to 4.81%)
During cesarean section	36 (14%)	37 (14%)	0.97 (0.64 to 1.49; 0.900)	0.38 (−5.47% to 6.22%)
**Chorioamnionitis**				
Histological chorioamnionitis^b^	43 (22%)	62 (32%)	0.69 (0.49 to 0.96; 0.026)	9.86% (1.22% to 18.5%)
Histological funisitis^b^	21 (11%)	34 (18%)	0.61 (0.36 to 1.004; 0.048)	6.99% (0.07% to 13.9%)
**Other complications**	16 (6.0%)	15 (5.6%)	1.07 (0.54 to 2.11; 0.853)	−0.38% (−4.36% to 3.61%)

Data are presented as number (percent).

a Percents, RRs, 95% CIs, and *p*-values given are related to available data per characteristic and may differ from the total number of patients.

b Outcome characteristic with more than 5% missing data. Histological chorioamnionitis data available for 396 women (74%); histological funisitis data available for 388 women (73%).

### Non-Randomized Women

Table 6 shows the baseline characteristics of the 207 non-randomized women. The non-randomized women differed to those randomized in level of education (more educated), smoking (fewer smoked), maternal age (older), and management (preferred EM). Furthermore, they differed in gestational age at PPROM (earlier PPROM). Neonatal sepsis was seen in one of 13 (7.7%) neonates born to mothers who preferred IoL, and in seven of the 198 (3.5%) neonates born to mothers who chose EM.

**Table 6:** Baseline characteristics for randomized versus non-randomized participants.

Characteristic^a^	Randomized (*n* = 532)	Non-Randomized (*n* = 207)	*p*-Value
**Maternal age (range) [±SD], y**	29.6 (18.0–46.7) [±5.3]	32.3 (19.5–45.5) [±5.0]	<0.0001
**Number nulliparous (parity range) (percent nulliparous)**	299 (0–5) (56%)	121 (0–3) (58%)	0.580
**Twin pregnancy**	6 (1.1%)	4 (1.9%)	0.395
**Ethnic origin**			
White	420 (79%)	149 (72%)	
Other ethnic origin	78 (15%)	37 (18%)	0.188
Unknown	34 (6.4%)	21 (10%)	NA
**Education** b **^,^** c			
Primary school (4 to 12 y)	10 (3.2%)	0 (0%)	
Secondary school (12–18 y)^b^	26 (8.4%)	10(8.7%)	
Lower professional school	29 (9.4%)	5 (4·4%)	
Medium professional school	149 (48%)	42 (37%)	
Higher professional school	70 (23%)	37 (32%)	
University	26 (8.4%)	21 (18%)	0.002
**Maternal smoking**	121 (24%)	21 (11%)	0.001
**Body mass index** c			
At booking (range) [±SD], kg/m^2^	24.7 (16.4–52.2) [±5.4]	24.4 (17.3–41.9) [±4.5]	0.605
At study entry (range) [±SD], kg/m^2^	29.1 (16.3–52.1) [±6.0]	28.0 (17.3–43.8) [±4.7]	0·139
**Gestational age at PPROM**			
<34 wk	74 (14%)	60 (29%)	
34^+0^ to 34^+6^ wk	76 (14%)	38 (18%)	
35^+0^ to 35^+6^ wk	163 (31%)	64 (31%)	
36^+0^ to 36^+6^ wk	219 (41%)	44 (21%)	<0.0001
**Gestational age at PPROM, median [IQR], d**	249 [243–253]	244 [234–250]	<0.0001
**Maternal temperature at inclusion, mean [±SD],°C** c	36.9 [±0.46]	36.8 [±0.46]	0.214
**Treatment**			
IoL	266 (50%)	13 (6.3%)	
EM	266 (50%)	194 (94%)	<0.0001

Data are presented as number (percent) unless otherwise indicated.

a Percents given are related to available data per characteristic and may differ from the total number of patients.

b Percents given as part of known educational level.

c Outcome characteristic with more than 5% missing data. Education: data available for 425 women (58%); body mass index at booking: data available for 616 women (83%); body mass index at start study available for 346 women (47%); maternal temperature at inclusion: data available for 689 women (93%).

### Meta-Analysis

**Figure 3:**
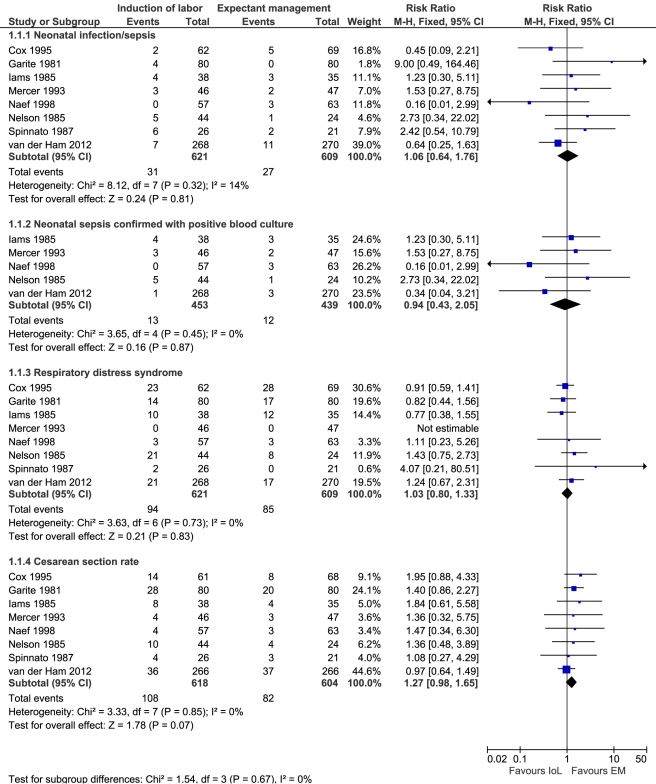
Meta-analysis. Risk ratio according to Mantel-Haenszel (M–H) with fixed effects and 95% CIs for neonatal sepsis, culture-proven neonatal sepsis, RDS, and cesarean section rates.

The electronic search yielded ten new results relevant for meta-analysis. After reviewing these papers, none fulfilled the inclusion criteria. Figure 3 presents the results of the meta-analysis including our own data. In total, 1,230 neonates could be analyzed from eight studies for neonatal sepsis, 892 neonates (five studies) for culture-proven sepsis, 1,230 neonates (eight studies) for RDS, and 1,222 women (eight studies) for cesarean section rate. None of the risk ratios for these outcomes were statistically different (1.06 [95% CI 0.64–1.76], *p* = 0.81; 0.94 [95% CI 0.43–2.05], *p* = 0.87; 1.03 [95% CI 0.80–1.33], *p* = 0.83; 1.27 [95% CI 0.98 to 1.65], *p* = 0.07, respectively).

## Discussion

We conducted a large randomized study (the PPROMEXIL trial) to compare IoL and EM in women with PPROM between 34 and 37 wk of gestational age. Because of the conservative treatment policy and conservative preferences amongst patients in The Netherlands, we had an ideal population in which to perform this trial, with extensive data on all eligible patients including non-participants (31%), the vast majority of whom declined participation because they preferred EM.

We found that in pregnancies complicated by PPROM between 34 and 37 wk of gestation, IoL does not substantially reduce the incidence of neonatal sepsis compared to EM. The number of neonates with respiratory distress was comparable in both arms, and IoL did not increase the risk of a cesarean section, findings that were confirmed in meta-analysis. However, in our study IoL increased the risk of hypoglycemia and hyperbilirubinemia, as well as the use of epidural or spinal analgesia during labor.

Our findings are in line with the results of the TERMPROM trial, which compared IoL with EM in 5,041 women with prelabor rupture of membranes at term [17]. The TERMPROM trial showed that IoL did not reduce the risk of neonatal sepsis as compared to EM (2.5% versus 2.8%) [17].

In contrast to earlier studies [15],[17]–[19], our pragmatic protocol did not include routine cultures from all neonates to diagnose sepsis. Because of the lack of consensus amongst Dutch neonatologists on whether to take blood samples routinely after prolonged premature rupture of membranes, neonatal blood samples and liquor cultures were taken only on clinical indication. All cases with any possible sign of neonatal sepsis were adjudicated by a panel of neonatologists. In consensus they decided whether or not a newborn had suffered neonatal sepsis (suspected or proven). Despite of the lack of blood culture from all neonates in the trial, we believe that no cases of neonatal sepsis were missed and that the incidence of neonatal sepsis was not overestimated.

IoL reduced the risk of chorioamnionitis. Several studies have demonstrated a relationship between chorioamnionitis and adverse neonatal outcome [20]–[23]. In a large study of very premature neonates (<28 wk; the ELGAN study) [20], a relationship between cerebral palsy and/or white matter damage and positive bacteriological cultures from the placenta was demonstrated. Other studies have also described a relationship between chorioamnionitis and increased risk for sepsis, respiratory distress, pneumonia, and even neonatal death [22],[23]. We doubt, however, that these findings can be extrapolated to our population. The incidence of cerebral palsy is significantly lower in the near-term-birth population, and reported incidences of adverse neonatal outcome in a near-term and term newborns are low (maximum reported incidences of 1.9% in the chorioamnionitis group) [23].

In line with the TERMPROM trial, the number of cesarean sections in our study was comparable in the IoL and EM groups [17]. We could not confirm the trend for increased risk on cesarean section in the EM group that was reported in the previous systematic review [7].

The risks of hypoglycemia and hyperbilirubinemia were decreased in children of women treated expectantly. These findings have, to our knowledge, not previously been reported for a prospective study. In a recent retrospective study in three tertiary hospitals in France, a similar incidence of hyperbilirubinemia (37% for IoL versus 33% for EM) and a slightly lower incidence of hypoglycemia (12% for IoL versus 6% for EM) was found, but due to a lack of power, differences were not statistically significant in the French study [24].

We doubt whether prolongation of pregnancy, which was on average 3.3 d longer in the EM group in our trial, will have solely contributed to the decreased risk of hypoglycemia and hyperbilirubinemia. Maybe spontaneous onset of labor enhances the speed of physiological maturing by means of a still unknown adaptational process, as is known to happen in the lungs, reducing the incidence of RDS in spontaneous delivery compared to elective cesarean section [25].

The clinical importance of these findings for later (cognitive and motor) development in children is not clear at present for our study group, although it is known that following symptomatic neonatal hypoglycemia, more than 50% of infants demonstrate cognitive and motor impairments at the age of 18 mo [26]. In low-birth-weight infants, even an asymptomatic moderate hypoglycemia may lead to cognitive and motor impairments at the age of 18 mo [27]. Hyperbilirubinemia is potentially neurotoxic, especially in infants born preterm [28]. When treated appropriately, however, bilirubin levels under 30 mg/dl are not associated with adverse neurodevelopmental outcome [29].

MacKay et al. [30] reported on the increased need for special education in preterm-delivered infants. In a retrospective cohort study of 407,503 schoolchildren, they showed that gestational age at delivery had a strong, dose-dependent relationship with special educational need. Until further evidence becomes available, the decreased risk of special educational need with advancing gestational age should be taken into account when considering how best to manage PPROM.

Within the Dutch Consortium for Women's Health and Reproductivity Studies (http://www.studies-obsgyn.nl), the PPROMEXIL trial is the largest trial so far with regard to the number of participating hospitals (60 out of 98 eligible hospitals, 61%). The 207 non-randomized women in our study who allowed data collection differed from the randomized women. Similar to two other Dutch consortium trials (HYPITAT and DIGITAT) [31],[32], the women who agreed to be randomized differed in level of education, smoking habits, maternal age, and preferred management from those who did not agree to be randomized. The non-randomized subgroup of women who preferred IoL was too small to draw any conclusions from. In the EM subgroup, no differences were seen in the primary outcome or the secondary neonatal and maternal outcomes. Even though some women eligible to participate in the trial did not, we believe that we did not miss a significant group at a higher (or lower) risk for neonatal sepsis who were treated expectantly. Despite some differences in baseline characteristics, we assume that the results of our study can be generalized to at least the Dutch/Western European population. Because of wide differences in general health care and availability of antibiotics, it is likely that these results cannot be generalized to low-income countries.

The main limitation of our study is that it proved to be underpowered. We hypothesized a decrease in neonatal sepsis rate of 7.5% to 2.5%, but found a difference of only 1.5% (2.6% in the IoL group versus 4.1% in the EM group). The liberal use of antibiotic therapy before or during labor (41% of all participating women received antibiotics) might have contributed to a lower incidence compared to the other trials in which antibiotics were not administered prophylactically [15],[18],[33]–[37]. These previous studies showed high rates of neonatal sepsis with EM. Similarly, improved health care may have contributed to a reduction of the incidence of neonatal sepsis in women with PPROM over the last decades.

In our study the observed difference in sepsis rates between the EM and IoL groups did not reach statistical significance. Although this study is one of the largest published so far, our sample size was insufficient to demonstrate small differences. In retrospect, anticipating a risk reduction of 66% (a difference in neonatal sepsis rate of 7.5% versus 2.5%) might have been too optimistic. However, several previous studies [18],[33],[35] showed neonatal sepsis incidences up to 9.5% with EM, and we did observe an incidence near 2.5% in the IoL group. Although optimistic, we feel that our hypothesized risk reduction was not unrealistic. Furthermore, we used a one-sided test for the power calculation. We considered it not plausible that IoL in women with PPROM near term would increase the proportion of cases of neonatal sepsis. In retrospect, considering the results of the meta-analysis, one might question this choice of a one-sided test, as several studies in the meta-analysis show an increased risk for sepsis in the IoL group. However, the analysis was executed exactly as planned in advance. Two-sided testing would have required a sample size that would not have been feasible in our setting, in view of the limitations set by our funding body.

A second potential limitation is that EM prolonged gestation by just 4 d. This rather small difference might be partly due to the fact that the median gestational age at rupture of membranes was 35^+4^ wk and median gestational age at randomization was 35^+6^ wk. The overrepresentation of women beyond the 35^th^ completed week of gestation was caused by the fact that women in their 35^th^ week of gestation more often refused to participate (mean gestational age at PPROM in the non-randomized group was 34^+6^ wk), and before our study an expectant policy was the standard. Furthermore, hesitation of clinicians to induce labor before 35 completed weeks of gestation, which prior to the start of the PPROMEXIL trial was not recommended in the Dutch guidelines, might also have influenced this outcome.

A third limitation of this study is that we reported many secondary, mostly neonatal, outcomes. Although this is not uncommon in studies in maternal–fetal medicine, it is possible that a significant difference can be found by chance. If one applies a Bonferroni correction to the *p*-value, the adjusted threshold is *p*<0.001. By applying this threshold, the incidence of hypoglycemia (*p* = 0.0008) remains the only statistically significant difference between the groups.

We are aware of the ongoing multicenter PPROMT trial (ISRCTN44485060), which has a design similar to that of our study. That trial may possibly answer the question whether IoL in women with near-term PPROM reduces the risk of neonatal sepsis [38]. However, the updated meta-analysis clearly demonstrates that the incidence of neonatal sepsis is comparable in both treatment strategies [38]. We therefore plan to perform an individual patient data meta-analysis on the management of PPROM. Combining large trials in an individual patient data meta-analysis would, in our opinion, produce the best currently available evidence for the management of PPROM. We have already planned such an analysis with the PPROMT study group, and will contact researchers from other published trials to collaborate in an individual patient data meta-analysis.

We conclude that in pregnancies complicated by PPROM between 34 and 37 wk of gestation the incidence of neonatal sepsis is low. Neither our trial nor the updated meta-analysis shows that IoL substantially improves pregnancy outcomes compared with EM.

## Supporting Information

Text S1 Study protocol. Previously published in [13].
(PDF)

Text S2 CONSORT statement.
(DOC)

## Abbreviations

PPROM: preterm prelabor rupture of membranes
PPROMEXIL: PPROM Expectant Management versus Induction of Labor
CI: confidence interval
EM: expectant management
HELLP syndrome: hemolysis, elevated liver enzymes, and low platelets
IoL: induction of labor
NICU: neonatal intensive care unit
RDS: respiratory distress syndrome
RR: relative risk
