# Supplementary material for: Induction of Labor versus Expectant Management in Women with Preterm Prelabor Rupture of Membranes between 34 and 37 Weeks: A Randomized Controlled Trial
Source: PLoS Med. 2012 Apr 24;9(4):e1001208. doi: 10.1371/journal.pmed.1001208 (PMC3335867; doi:10.1371/journal.pmed.1001208)
Supplement: Text S1 — Study protocol. Previously published in [13]. (PDF) [file pmed.1001208.s001.pdf]

BMC Pregnancy and Childbirth

BioMed Central

Study protocol

Open Access

Induction of labour versus expectant management in women with preterm prelabour rupture of membranes between 34 and 37 weeks (the PPROMEXIL-trial)

David P van der Ham*[1], Jan G Nijhuis[2], Ben Willem J Mol[3], Johannes J van Beek[1], Brent C Opmeer[4], Denise Bijlenga[5], Mariette Groenewout[6], Birgit Arabin[7], Kitty WM Bloemenkamp[8], Wim J van Wijngaarden[9], Maurice GAJ Wouters[10], Paula JM Pernet[11], Martina M Porath[3], Jan FM Molkenboer[12], Jan B Derks[13], Michael M Kars[14], Hubertina CJ Scheepers[15], Martin JN Weinans[16], Mallory D Woiski[15], Hajo IJ Wildschut[17] and Christine Willekes[2]

Address: [1]Department of Obstetrics and Gynaecology, VieCuri Medical Centre Venlo, the Netherlands, [2]Department of Obstetrics and Gynaecology, University Hospital Maastricht, the Netherlands, [3]Department of Obstetrics and Gynaecology, Maxima Medical Centre Veldhoven, the Netherlands, [4]Department of Clinical Epidemiology, Biostatistics and Bioinformatics, Academic Medical Centre Amsterdam, the Netherlands, [5]Department of Social Medicine, Academic Medical Centre Amsterdam, the Netherlands, [6]Department of Obstetrics and Gynaecology, University Medical Centre Groningen, the Netherlands, [7]Department of Obstetrics and Gynaecology Isala Klinieken Zwolle, the Netherlands, [8]Department of Obstetrics and Gynaecology, University Medical Centre Leiden, the Netherlands, [9]Department of Obstetrics and Gynaecology, Bronovo Hospital the Hague, the Netherlands, [10]Department of Obstetrics and Gynaecology, VU Medical Centre Amsterdam, the Netherlands, [11]Department of Obstetrics and Gynaecology, Kennemer Gasthuis Haarlem, the Netherlands, [12]Department of Obstetrics and Gynaecology, Sint Anna Hospital Geldrop, the Netherlands, [13]Department of Obstetrics and Gynaecology, University Medical Centre Utrecht, the Netherlands, [14]Department of Obstetrics and Gynaecology, Mesos Medical Centre Utrecht, the Netherlands, [15]Department of Obstetrics and Gynaecology, University Medical Centre Sint Radboud Nijmegen, the Netherlands, [16]Department of Obstetrics and Gynaecology, Gelderse Vallei Hospital Ede, the Netherlands and [17]Department of Obstetrics and Gynaecology, Erasmus Medical Centre Rotterdam, the Netherlands

Email: David P van der Ham* - dpvanderham@zonnet.nl; Jan G Nijhuis - jnij@sgyn.azm.nl; Ben Willem J Mol - b.w.mol@amc.uva.nl; Johannes J van Beek - beekjj@xs4all.nl; Brent C Opmeer - b.c.opmeer@amc.uva.nl; Denise Bijlenga - d.bijlenga@amc.uva.nl; Mariette Groenewout - m.vanderzwet@tiscali.nl; Birgit Arabin - clara-angela@wxs.nl; Kitty WM Bloemenkamp - k.w.m.bloemenkamp@lumc.nl; Wim J van Wijngaarden - m.vanwijngaarden@chello.nl; Maurice GAJ Wouters - mgaj.wouters@vumc.nl; Paula JM Pernet - pernet@kg.nl; Martina M Porath - m.porath@mmc.nl; Jan FM Molkenboer - jfmm@xs4all.nl; Jan B Derks - jbderks@hotmail.com; Michael M Kars - mmkars@freeler.nl; Hubertina CJ Scheepers - L.Scheepers@obgyn.umcn.nl; Martin JN Weinans - weinansm@zgv.nl; Mallory D Woiski - M.Woiski@obgyn.umcn.nl; Hajo IJ Wildschut - h.wildschut@erasmusmc.nl; Christine Willekes - cwi@sgyn.azm.nl

* Corresponding author

Published: 6 July 2007

BMC Pregnancy and Childbirth 2007, **7**:11 doi:10.1186/1471-2393-7-11

Received: 29 March 2007
Accepted: 6 July 2007

This article is available from: http://www.biomedcentral.com/1471-2393/7/11

© 2007 van der Ham et al; licensee BioMed Central Ltd.
This is an Open Access article distributed under the terms of the Creative Commons Attribution License (http://creativecommons.org/licenses/by/2.0), which permits unrestricted use, distribution, and reproduction in any medium, provided the original work is properly cited.

Abstract

Background: Preterm prelabour rupture of the membranes (PPROM) is an important clinical problem and a dilemma for the gynaecologist. On the one hand, awaiting spontaneous labour increases the probability of infectious disease for both mother and child, whereas on the other hand induction of labour leads to preterm birth with an increase in neonatal morbidity (e.g., respiratory distress syndrome (RDS)) and a possible rise in the number of instrumental deliveries.

Methods/Design: We aim to determine the effectiveness and cost-effectiveness of immediate delivery after PPROM in near term gestation compared to expectant management. Pregnant

women with preterm prelabour rupture of the membranes at a gestational age from 34^{+0} weeks until 37^{+0} weeks will be included in a multicentre prospective randomised controlled trial. We will compare early delivery with expectant monitoring.

The primary outcome of this study is neonatal sepsis. Secondary outcome measures are maternal morbidity (chorioamnionitis, puerperal sepsis) and neonatal disease, instrumental delivery rate, maternal quality of life, maternal preferences and costs. We anticipate that a reduction of neonatal infection from 7.5% to 2.5% after induction will outweigh an increase in RDS and additional costs due to admission of the child due to prematurity. Under these assumptions, we aim to randomly allocate 520 women to two groups of 260 women each. Analysis will be by intention to treat. Additionally a cost-effectiveness analysis will be performed to evaluate if the cost related to early delivery will outweigh those of expectant management. Long term outcomes will be evaluated using modelling.

Discussion: This trial will provide evidence as to whether induction of labour after preterm prelabour rupture of membranes is an effective and cost-effective strategy to reduce the risk of neonatal sepsis.

Controlled clinical trial register: ISRCTN29313500

Background

Preterm prelabour rupture of the membranes (PPROM) is an important clinical problem and a dilemma for the gynaecologist. On the one hand, awaiting spontaneous labour may lead to an increase in infectious disease for both mother and child, whereas on the other hand induction of labour leads to preterm birth with an increase in neonatal morbidity (e.g., respiratory distress syndrome (RDS)) and a possible rise in the number of instrumental deliveries.

The estimated incidence of PPROM between 34 and 37 weeks of gestation is 1.5%, which equals about 3.000 cases per year in the Netherlands. The incidence of RDS is estimated to decrease from 15% at 34 weeks to below 1% at 37 weeks' gestation [1]. On the other hand, the probability that sepsis occurs increases when expectant management is advocated. In case the child is born immediately after PPROM, the risk of sepsis is 2.5%, whereas it increases to 7.5% in case of expectant management [2,3].

Until now, management of PPROM between 34 and 37 weeks' gestation varies in the Netherlands. In the guideline of the Dutch Society of Obstetrics and Gynaecology (NVOG) expectant management is advocated if the gestational age is under 35 weeks [4]. Beyond 35 weeks, the guideline makes no clear recommendation, and the decision for either induction of labour or expectant management (either in hospital or with monitoring at home), is left to local protocols. International guidelines do not make a clear statement either [5,6].

This dilemma can be explained by a lack of good clinical evidence. As a consequence, the course of action on this subject varies in the Netherlands. Data from the Dutch National Delivery Registration indicate that in about 70% of the patients an expectant management has been practised, whereas in about 30% of the cases labour was induced preterm. The current national registrations provide no insight in the reason for induction in the latter group, as some inductions might have been performed following signs of intra-uterine infection.

In view of the lack of good clinical evidence and the practice variation described above, a randomised clinical trial comparing induction of labour and expectant monitoring in patients with PPROM is needed urgently.

We are aware of four small studies that have been performed to discern between the treatment policies for PPROM [7-10]. Naef et al. compared induction of labour versus expectant management in a group of 120 patients with PPROM occurring at 34 until 37 weeks gestation and found a non-significant decrease of chorioamnionitis in the induction group without a significant difference in neonatal morbidity (including RDS) between both groups [7]. Patients with PPROM managed expectantly were hospitalised significantly longer. More babies of the expectant group were diagnosed with sepsis and admitted to the neonatal ward for a longer duration. This difference did not reach the level of significance due to the small number of the study.

Cox et al. compared maternal and neonatal outcome in a group of 129 patients with PPROM occurring at 30 until 34 weeks' gestation after randomization between expectant management and induction of labour [8]. Again, no significant differences in neonatal outcome were noted. However, a non-significant decrease in sepsis was seen in the induction group. Also, chorioamnionitis was

observed less frequently in the latter group. Mercer et al. performed a similar study of 93 patients with PPROM in a different age category (32 until 36 weeks' gestation) and observed similar differences as Cox and co-workers [9].

Spinnato et al. found no difference in neonatal outcome between expected management and prompt delivery in 47 patients with premature rupture of membranes (before 36 weeks) with documented foetal pulmonary maturity [10]. However, they demonstrated an increased risk of maternal infection when expectant management was applied.

These studies show a trend towards better neonatal outcome in the induction group, but in these small samples differences were not statistically significant. Even meta-analysis did not generate sufficient statistical power [11]. In view of the practice variation described above and the lack of sound clinical evidence for this clinical dilemma, we have recently started a multicentre trial.

Methods/Design

Aims

The aim of this study is to systematically compare early initiation of delivery and expectant management in case of preterm prelabour rupture of membranes in terms of neonatal sepsis and RDS, maternal health, health-related quality-of-life and costs. As it is impossible to blind the healthcare workers and patients involved for the strategy of allocation, we will use a multicentre randomised controlled open label trial to assess the effects of initiation of delivery or expectant management on neonatal outcome. This study is set in the Dutch Obstetric Consortium, a collaboration of obstetric clinics in the Netherlands. Approximately 40 clinics, including academic hospitals, non-academic teaching hospitals and non-teaching hospitals will participate in this trial.

Participants/eligibility criteria

Women presenting with preterm prelabour rupture of the foetal membranes between 34^{+0} and 37^{+0} weeks' gestation who have not delivered within 24 hours after rupture of the foetal membranes are eligible for participation in the PPROMEXIL-trial. Even so women presenting with preterm prelabour rupture of foetal membranes after 26^{+0} weeks gestation who have not delivered at 34^{+0} weeks of gestation are eligible for participation. Both women with singleton and women with multiple gestations can be included. Women with a child in breech presentation can also be included, and both an elective caesarean section and a vaginal delivery are allowed for these women.

Women with monochorionic multiple pregnancies, abnormal (non-reassuring) cardiotocogram (CTG), meconium stained amniotic fluid, signs of intrauterine infection, major foetal anomalies, labour, HELLP syndrome or severe pre-eclampsia, will not be included in the study.

Procedures, recruitment, randomisation and collection of baseline data

The research nurse and/or the staff of participating hospitals will identify eligible women. After the subject has given informed consent for participation in the study, she will be randomised to either a policy that aims at termination of the pregnancy (intervention group) or a policy that aims at expectant management for spontaneous delivery (expectant group). Once the subject data have been entered anonymously in a web-based database, either a staff member or the research nurse will randomise using an internet based procedure. Randomisation will be 1:1 for intervention or expectant management and stratified for centre and previous delivery.

At study entry baseline demographics, past obstetric and medical history will be recorded. Maternal temperature is measured and baseline blood samples are taken. From all participating women a vaginal swab is collected either at admission to the hospital with premature ruptured membranes or at study entry. Antibiotics are given in accordance to local policy. All women will undergo an ultrasound examination at study entry. If a previous ultrasound examination was done within a fortnight before study entry in the same hospital, which revealed no abnormal results, these outcomes may be recorded at study entry. Women fill out a baseline quality of life questionnaire containing EuroQoL, 5D3L, HADS, SF-36 and background questions. They are also asked on their prior intervention preference.

At local centres data-collection will be the responsibility of the local research coordinator and the regional research nurses. The data for this study must be collected, coded and processed with adequate precautions to ensure patient confidentially.

Interventions

Intervention group

Women randomised for intervention will be planned for induction of labour or elective caesarean section as soon as reasonable after randomisation. Because of the fact that randomisation will take place preferable 24 hours after rupture of the foetal membranes it is imaginable that due to logistic reasons induction of labour is postponed until the following morning, but within 12 hours after randomisation. Induction of labour will take place according to local policy. In case of breech presentation, a primary caesarean section is allowed based on the preference of both the patient and the gynaecologist. Similarly in case of a dichorionic twin an elective caesarean section may be

performed because of the position of the presentation of the first twin.

Expectant group

Women randomised for expectant management will be treated according to local policy. This might be either in an outpatient or inpatient setting. If a patient in the expectant group reaches 37^{+0} weeks of gestation age, induction of labour is performed according to local policy. Whenever a patient with an indication for elective caesarean section will be allocated to expectant management the caesarean section will be performed as soon as labour commences.

Follow up of women and infants

Information is obtained on the condition, weight, length and neonatal morbidity and mortality from the infant and maternal records as well as maternal complications and length of stay. If applicable, neonatal antibiotic treatment, blood samples and cultures are recorded. In case of admittance of the baby to the neonatal intensive care, high care or medium care unit, details of this admittance are also documented. The placenta will be sent for histological examination on chorioamnionitis and funisitis.

Six weeks and six months post partum neonatal length, weight, neurological disabilities, physical disabilities and maternal disabilities will be recorded. Patients will be asked to fill out follow-up quality of life questionnaires including EuroQoL, 5D3L, HADS, SF-36, SCL-90 also concerning the development of the child. Women are also asked to (re)state their intervention preference. Long-term follow up of the children is not yet planned, and depends on future funding.

Outcome measures

The primary outcome measure is neonatal sepsis. Neonatal sepsis is defined as a positive blood culture, biochemical infection parameters (C-reactive protein (CRP) above 20 mg/l) or clinical signs of infection (apnoea, fever, intolerance for feeding, respiratory distress and/or haemodynamic instability) with positive surface cultures. An independent panel of paediatricians will define between proven or probable sepsis without knowledge of the outcome data.

Secondary infant outcome measures are respiratory distress syndrome (RDS) (defined according to the Organ dysfunction criteria), transient tachypnoea of the newborn asphyxia (defined according to Sarnat), pneumothorax/pneumomediastinum, late onset sepsis, hypoglycaemia, meconium aspiration syndrome, necrotizing enterocolitis (NEC) (defined according to Bell staging), hyperbilirubinemia, in, traventricular haemorrhage, periventricular leucomalacia, convulsions, other neurological abnormalities and congenital abnormalities [12-14].

Secondary maternal outcome measures are ante partum haemorrhage, umbilical cord prolapse, signs of chorioamnionitis (defined as fever before or during labour as a temperature greater than 37,5°C on two occasion more than one hour apart or a temperature > 38,0°C with either uterine tenderness (or contractions), leucocytosis, maternal or foetal tachycardia, or a foul-smelling vaginal discharge in absence of any other cause of hyperpyrexia), maternal sepsis (defined as temperature > 38.5°C and positive blood culture or circulatory instability requiring intensive care monitoring), thrombo-embolic complications, urinary tract infection treated with antibiotics, signs of endometritis (defined as temperature of > 38,0 °C on 2 occasions at least one our apart after the first 24 h postpartum with associated uterine tenderness [15]), pneumonia, anaphylactic shock, HELLP-syndrome and death, incidence of instrumental deliveries, maternal quality of life, maternal intervention preference and costs. [15]

Other outcomes are direct medical and non-medical costs generated by maternal and neonatal resource utilisation during admission and post-discharge follow-up until 6 weeks after randomisation. The economic evaluation will integrate the primary clinical outcome and costs in a cost-effectiveness analysis.

Statistical analysis

Sample size

This trial is designed to demonstrate a 66% reduction of neonatal sepsis from 7.5% in women in the expectant group to 2.5% in women in the induction group. This requires a total sample size of 520 patients with 80 % power and a significance level of $p = 0.05$.

Data analysis

Data analysis will be performed on an intention to treat basis and study baseline characteristics will be compared. The relative risks and 95% confidence intervals will be calculated for the relevant outcome measures. The analysis will be stratified for centre and parity. Moreover, we will evaluate whether the relative benefits of induction of labour will be stronger in multiparous women and in women with a ripe cervix at baseline.

In case of continuous data in the secondary outcome group, t-tests will be used whereas chi-square tests will be used if data are categorical. In case of equivalence between outcomes, the analysis will be repeated on a par protocol basis. Quality of life as well as pain scores will be analysed using repeated measures analysis of variance. [16]

Serious Adverse Events will be reported to an independent data safety monitoring committee. A formal interim analysis is not planned.

Ethical considerations

This study has been approved by the ethic committee of the University Hospital Maastricht (Ref. no. MEC 05–240). A total of about 40 academic and non-academic hospitals have signed an intention form to participate in the trial, most of them have started the medical ethics committee approval procedure. Before a collaboration clinic may start with randomisation of patients the local ethical committee must have given their approval. The trial is registered in the controlled clinical trial register under number: ISRCTN29313500 [17]

Confidentially and data security

Initials of participants as well as a local patient number are recorded in the electronic database. Linking names with patient's numbers can only be done in the local clinics. Data will be collected using Oracle Clinical Remote Data Capture (RDC), which is a new generation of application system that enables collection and cleanup of clinical trial data using the Internet. For detailed information on Oracle RDC, please visit the page of Oracle RDC products. Each participating clinic receives a login name and password to get access to the web-secured database. The access is restricted to the database of the clinic to which the password and login name belongs. Full access to the entire database is restricted to some members of the research staff.

Discussion

Preterm prelabour rupture of membranes is still a clinical problem in obstetric practice. Between 34 and 37 weeks there is a lack of good clinical evidence. Induction of labour might reduce the risks of neonatal sepsis. On the other hand it might increase the incidence of instrumental deliveries and neonatal respiratory distress syndrome. This trial is designed to answer these questions in respect to neonatal and maternal outcomes and costs-effectiveness.

We are aware of two other studies on the subject that are running. In Canada, a trial has been started that compares induction of labour to expectant management in women with PPROM. [18] This trial aims to recruit 360 women (Safety and Efficacy Study of Intentional Delivery in Women With Preterm and Prelabour Rupture of the Membranes, Lacaze N).

In St Leonards, Australia, a trial has been recently started similar to the trial that we have started (PPROMT – Preterm Prelabour Rupture Of the Membranes close to Term) [19]. This trial aims to recruit 1812 women and has been announced in the journal [20]. The respective investigator groups, including ours, have planned an individual patient data meta-analysis after completion of the trials.

Competing interests

The author(s) declare that they have no competing interests.

Authors' contributions

DH drafted the manuscript; DH, BM, DB, and CW participated in the design of the study; BO participated in the design of the statistical analysis. All authors discussed and fine tuned the final study design. All authors are participating in the acquisition of data. Finally all authors read, revised and approved the final manuscript.

Acknowledgements

This study is funded by ZonMW (grant number 945-07-212). [21]

References

1. Lewis DF, Futayyeh S, Towers CV, Asrat T, Edwards MS, Brooks GG: **Preterm delivery from 34 to 37 weeks of gestation: Is respiratory distress syndrome a problem?** *Am J Obstet Gynecol* 1996, **174:**525-528.
2. Neerhof MG, Cravello C, Haney EI, Siver RK: **Timing of labour induction after premature rupture of membranes between 32 and 36 weeks gestation.** *Am J Obstet Gynecol* 1999, **180:**349-352.
3. Lieman JM, Brumfield CG, Carlo W: **Preterm premature rupture of membranes: Is there an optimal gestational age for delivery?** *Obstet Gynecol* 2005, **105:**12-17.
4. **NVOG richtlijn no. 46; Het breken van de vliezen voor het begin van de baring; juni.** 2002.
5. American College of Obstetricians and Gynecologists: **Premature rupture of membranes.** In *ACOG Practice Bullitin 1* Washington DC: ACOG; 1998.
6. Garite TJ: **Management of premature rupture of membranes.** *Clin Perinatol* 2001, **28:**837-844.
7. Naef RW, Albert A, Ross EL, Weber M, Martin RW, Morrison : **Premature rupture of membranes at 34 to 37 weeks gestation: Aggressive versus conservative management.** *Am J Obstet Gynecol* 1998, **178:**126-130.
8. Cox SM, Leveno KJ: **Intentional delivery versus expectant management with preterm premature ruptured membranes at 30 – 34 weeks gestation.** *Obstet Gynecol* 1995, **86:**875-879.
9. Mercer BM, Crocker LG, Boe NM, Sibai MB: **Induction versus expectant management in premature rupture of the membranes with mature amniotic fluid at 32 to 36 weeks: a randomized trial.** *Am J Obstet Gynecol* 1993, **169:**775-782.
10. Spinnato JA, Shaver DC, Bray EM, Lipshitz J: **Preterm premature rupture of the membranes with fetal pulmonary maturity present: a prospective study.** *Obstet Gynecol* 1987, **69:**96-201.
11. Hartling L, Chari R, Friesen C, Vandermeer B, Lacaze-Masmonteil T: **A systematic review of intentional delivery in women with preterm prelabor rupture of membranes.** *J Matern Fetal Neonatal Med* 2006, **19:**177-87.
12. Goldstein B, Giroir B, Randolph A: **International Consensus Conference on Pediatric Sepis. International pediatric sepsis consensus conference: Definitions for sepsis and organ dysfunction in pediatrics.** *Pediatr Crit Care Med* 2005, **6:**2-8.
13. Sarnat HB, Sarnat HS: **Neonatal encephalopathy following fetal distress: A clinical and EEG study.** *Arch Neurol* 1976, **33:**696-705.
14. Neu J: **Necrotizing enterocolitis: the search for a unifying pathogenic theory leading to prevention.** *Pediatr Clin North Am* 1996, **43:**409-432.
15. Lieman JM, Brumfield CG, Carlo W, Ramsey PS: **Preterm premature rupture of membranes: Is there an optimal gestational age for delivery?** *Obstet Gynecol* 2005, **105:**12-17.

16. Hays WL: *Statistics* 4th edition. New York: Holt, Rinehart and Winston; 1998.
17. **mRCT – Preterm Premature Rupture Of Membranes between 34 and 37 weeks: EXpectant management versus Induction of Labour** [http://www.controlled-trials.com/mrct/trial/257003/PPROMEXIL]
18. **Clinical trial: Safety and Efficacy Study of Intentional Delivery in Women With Preterm and Prelabour Rupture of the Membranes** [http://clinicaltrials.gov/ct/show/NCT00259519;jsessionid=511720C5DF99149F8A22D293DBDF071E?order=1]
19. **mRCT – A randomised controlled trial of immediate delivery versus expectant care in women with ruptured membranes close to term** [http://www.controlled-trials.com/mrct/trial/228805/PPROMT]
20. Morris JM, Roberts CL, Crowther CA, Buchanan SL, Henderson-Smart DJ, Salkeld G: **Protocol for the immediate delivery versus expectant care of women with preterm prelabour rupture of the membranes close to term (PPROMT) Trial [ISRCTN44485060].** *BMC Pregnancy Childbirth* 2006, **6**:9.
21. **ZonMw-Projectenpoort – Induction of labour versus expectant management in women with preterm premature rupture of membranes between 34 and 37 weeks** [http://zonmw.collexis.net/projectsummary.asp?foreignid=94507212]

Pre-publication history

The pre-publication history for this paper can be accessed here:

http://www.biomedcentral.com/1471-2393/7/11/prepub

Publish with **BioMed Central** and every scientist can read your work free of charge

"BioMed Central will be the most significant development for disseminating the results of biomedical research in our lifetime."

Sir Paul Nurse, Cancer Research UK

Your research papers will be:

- available free of charge to the entire biomedical community
- peer reviewed and published immediately upon acceptance
- cited in PubMed and archived on PubMed Central
- yours — you keep the copyright

Submit your manuscript here:
http://www.biomedcentral.com/info/publishing_adv.asp

BioMedcentral
