# Supplementary material for: Induction of Labor versus Expectant Management in Women with Preterm Prelabor Rupture of Membranes between 34 and 37 Weeks: A Randomized Controlled Trial
Source: PLoS Med. 2012 Apr 24;9(4):e1001208. doi: 10.1371/journal.pmed.1001208 (PMC3335867; doi:10.1371/journal.pmed.1001208)
Supplement: Text S2 — CONSORT statement. (DOC) [file pmed.1001208.s002.doc]

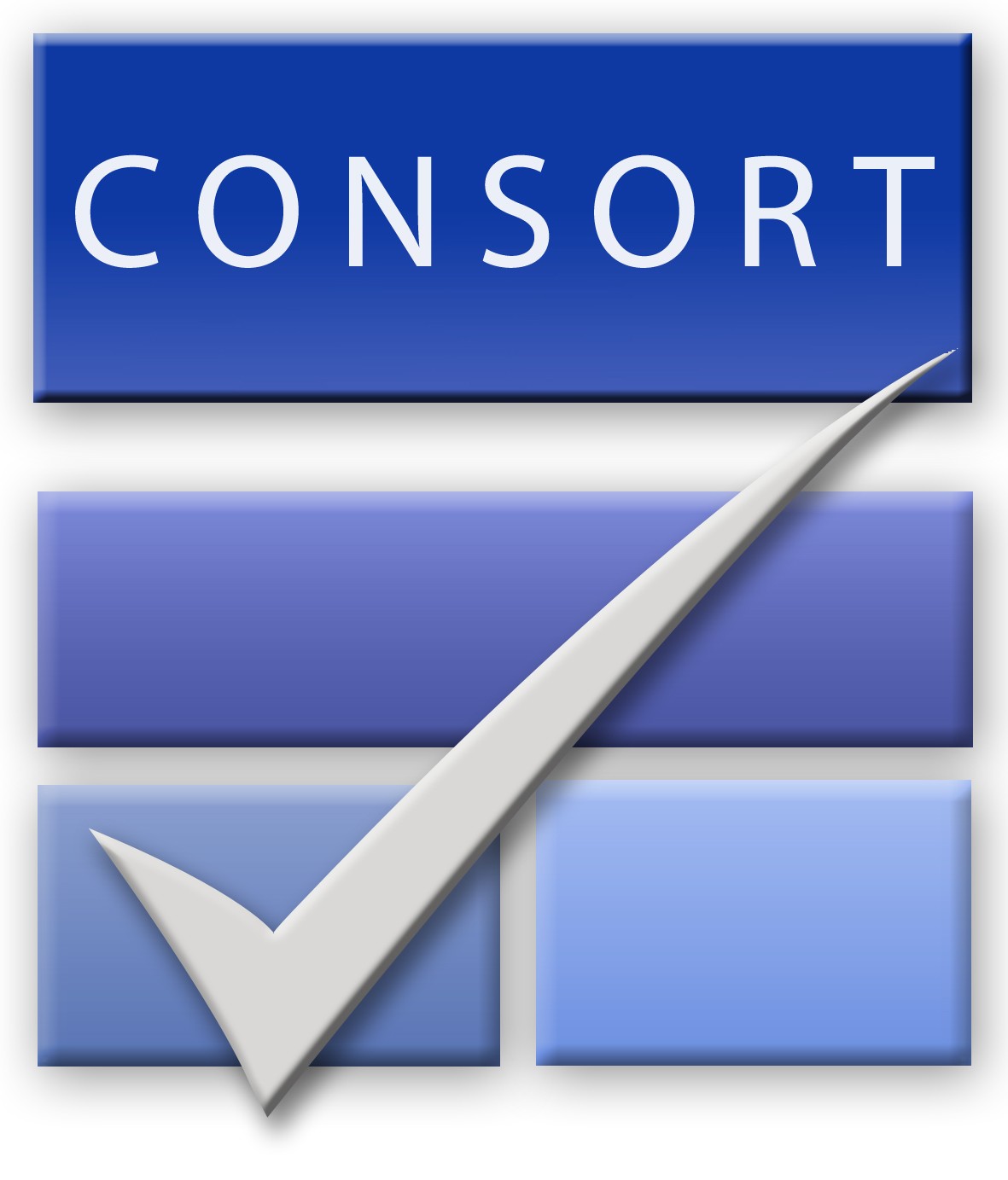
CONSORT 2010 checklist of information to include when reporting a randomised trial*

| Section/Topic | Item No | Checklist item | | Reported on page No |
| --- | --- | --- | --- | --- |
| Title and abstract | | | | |
|  | 1a | Identification as a randomised trial in the title | | Title |
|  | 1b | Structured summary of trial design, methods, results, and conclusions (for specific guidance see CONSORT for abstracts) | | Abstract section |
| Introduction | | | | |
| Background and objectives | 2a | Scientific background and explanation of rationale | | Introduction section |
|  | 2b | Specific objectives or hypotheses | | Introduction section, § 4 |
| Methods | | | | |
| Trial design | 3a | Description of trial design (such as parallel, factorial) including allocation ratio | Methods section, § 1 | |
|  | 3b | Important changes to methods after trial commencement (such as eligibility criteria), with reasons | Methods section, § 1 | |
| Participants | 4a | Eligibility criteria for participants | Methods section, patients | |
|  | 4b | Settings and locations where the data were collected | Methods section, § 1 | |
| Interventions | 5 | The interventions for each group with sufficient details to allow replication, including how and when they were actually administered | Methods section, Procedures | |
| Outcomes | 6a | Completely defined pre-specified primary and secondary outcome measures, including how and when they were assessed | Methods section, Outcome measures | |
|  | 6b | Any changes to trial outcomes after the trial commenced, with reasons | Methods section, § 1 | |
| Sample size | 7a | How sample size was determined | Methods section, Statistical analysis, Sample size | |
|  | 7b | When applicable, explanation of any interim analyses and stopping guidelines | Methods section, Data collection, § 4 | |
| Randomisation: |  |  |  | |
| Sequence generation | 8a | Method used to generate the random allocation sequence | Methods section, Randomization | |
|  | 8b | Type of randomisation; details of any restriction (such as blocking and block size) | Methods section, Randomization | |
| Allocation concealment mechanism | 9 | Mechanism used to implement the random allocation sequence (such as sequentially numbered containers), describing any steps taken to conceal the sequence until interventions were assigned | Methods section, Randomzation | |
| Implementation | 10 | Who generated the random allocation sequence, who enrolled participants, and who assigned participants to interventions | Methods section, Randomization and Data collection, § 1 | |
| Blinding | 11a | If done, who was blinded after assignment to interventions (for example, participants, care providers, those assessing outcomes) and how | N/A | |
|  | 11b | If relevant, description of the similarity of interventions | N/A | |
| Statistical methods | 12a | Statistical methods used to compare groups for primary and secondary outcomes | Methods section, Statistical analysis, Data analysis | |
|  | 12b | Methods for additional analyses, such as subgroup analyses and adjusted analyses | N/A | |
| Results | | | | |
| Participant flow (a diagram is strongly recommended) | 13a | For each group, the numbers of participants who were randomly assigned, received intended treatment, and were analysed for the primary outcome | | Results section, § 1 |
|  | 13b | For each group, losses and exclusions after randomisation, together with reasons | | Figure #1 |
| Recruitment | 14a | Dates defining the periods of recruitment and follow-up | | Results section, § 1 |
|  | 14b | Why the trial ended or was stopped | | Results section, § 1 |
| Baseline data | 15 | A table showing baseline demographic and clinical characteristics for each group | | Table #1 |
| Numbers analysed | 16 | For each group, number of participants (denominator) included in each analysis and whether the analysis was by original assigned groups | | Table #2, #3, #4 and #5 |
| Outcomes and estimation | 17a | For each primary and secondary outcome, results for each group, and the estimated effect size and its precision (such as 95% confidence interval) | | Table #3 |
|  | 17b | For binary outcomes, presentation of both absolute and relative effect sizes is recommended | | Table #2, #3, #4 and #5 |
| Ancillary analyses | 18 | Results of any other analyses performed, including subgroup analyses and adjusted analyses, distinguishing pre-specified from exploratory | | Results section, Neonatal sepsis |
| Harms | 19 | All important harms or unintended effects in each group (for specific guidance see CONSORT for harms) | | Results section, § 4 |
| Discussion | | | | |
| Limitations | 20 | Trial limitations, addressing sources of potential bias, imprecision, and, if relevant, multiplicity of analyses | | Discussion section, § 11-§13 |
| Generalisability | 21 | Generalisability (external validity, applicability) of the trial findings | | Discussion section, § 10 |
| Interpretation | 22 | Interpretation consistent with results, balancing benefits and harms, and considering other relevant evidence | | Discussion section, § 2- §9, §14 |
| Other information | | | |  |
| Registration | 23 | Registration number and name of trial registry | | Abstract section, Trial registration |
| Protocol | 24 | Where the full trial protocol can be accessed, if available | | Reference #13 AND Text S1 – Study Protocol |
| Funding | 25 | Sources of funding and other support (such as supply of drugs), role of funders | | Abstract section, Funding |

*We strongly recommend reading this statement in conjunction with the CONSORT 2010 Explanation and Elaboration for important clarifications on all the items. If relevant, we also recommend reading CONSORT extensions for cluster randomised trials, non-inferiority and equivalence trials, non-pharmacological treatments, herbal interventions, and pragmatic trials. Additional extensions are forthcoming: for those and for up to date references relevant to this checklist, see [www.consort-statement.org](http://www.consort-statement.org/).
